# SETD2-H3K36ME3: an important bridge between the environment and tumors

**DOI:** 10.3389/fgene.2023.1204463

**Published:** 2023-06-09

**Authors:** Jiahui He, Tangpeng Xu, Fangrui Zhao, Jin Guo, Qinyong Hu

**Affiliations:** Department of Oncology, Renmin Hospital of Wuhan University, Wuhan, Hubei, China

**Keywords:** SETD2 H3K36me3, tumor, environment, epigenetics, epigenetic drug

## Abstract

Epigenetic regulation plays an important role in the occurrence, development and treatment of tumors. The histone methyltransferase SET-domain-containing 2 (SETD2) plays a key role in mammalian epigenetic regulation by catalyzing histone methylation and interacting with RNA polymerase II to mediate transcription elongation and mismatch repair. As an important bridge between the environment and tumors, SETD2-H3K36me3 plays an important role in the occurrence and development of tumors. Many tumors, including renal cancer, gastric cancer, lung cancer, are closely related to *SETD2* gene mutations. As a key component of common tumor suppressor mechanisms, SETD2-H3K36me3is an important target for clinical disease diagnosis and treatment. Here, we reviewed the structure and function of the *SETD2* and how SETD2-H3K36me3 functions as a bridge between the environment and tumors to provide an in-depth understanding of its role in the occurrence and development of various tumors, which is of great significance for future disease diagnosis and treatment.

## 1 Introduction

Histone methylation is an important epigenetic modification that plays an important role in the occurrence, development and treatment of malignant tumors, which are a leading cause of death worldwide (Sung et al., 2021). The histone methyltransferase (HMT), SET-domain-containing 2 (SETD2) is an important member of the nuclear receptor SET domain (NSD) family and the only methyltransferase that catalyzes the formation of the H3K36me3 modification (Chen et al., 2020). SETD2-H3K36me3 is key component of common tumor suppressor mechanisms and an important target for cancer diagnosis and treatment (Leung et al., 2022). The main functions of SETD2/H3K36me3 are involved in DNA damage repair, maintaining active chromatin status, assisting transcription elongation, and thus promoting gene transcription levels. Tumor development is the result of the interaction of multiple risk factors, including those of environmental, exogenous and endogenous origins, as well as individual factors, including genetic susceptibility. Changes in the environment often cause epigenetic modifications that are reflected accordingly in the state of the cell. Tumors also exhibit a close relationship with the epigenetic changes caused by environmental factors.

**FIGURE 1:**
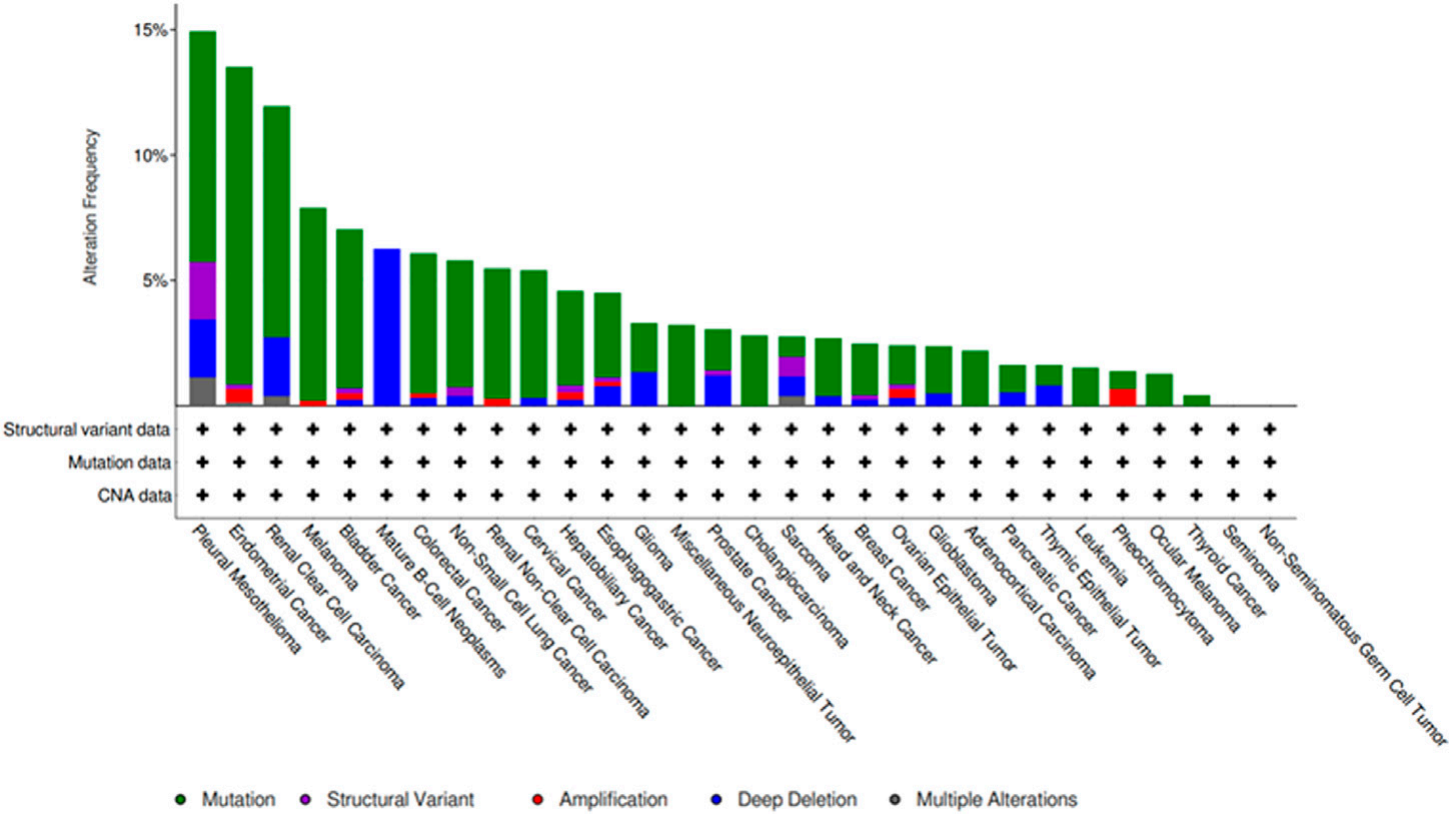
Frequency of *SETD2* mutations in different cancers from the cBioPortal database.

*SETD2* gene mutations or functional loss can cause protein dysfunction, leading to tumorigenesis, disease progression, chemotherapy resistance and poor prognosis. *SETD2* gene mutations have been identified in many cancers including kidney cancer (Cancer, 2013), pancreatic cancer (Niu et al., 2020a), prostate cancer (Yuan et al., 2020), leukemia (Dong et al., 2019), lung adenocarcinoma (Zhou Y et al., 2020), brain glioma (Fontebasso et al., 2013), breast cancer (Morcillo-Garcia et al., 2019), and gastrointestinal cancer (Chen et al., 2018). The cBioPortal database (https://www.cbioportal.org/) has also shown that SETD2 mutations occur in a variety of malignancies (Figure 1). SETD2-H3K36me3 is a common oncogenic mechanism in tumors, and its regulatory mechanism is tumor tissue specific. In this review, we first consider the structure and function of the *SETD2* and the relationship between tumors and epigenetic modifications, including DNA methylation, histone modification, chromatin remodeling and non-coding RNAs (ncRNAs). Next, we review the relationship between SETD2/H3K36me3 and most tumors, and describe in detail the antitumor function of SETD2 in a variety of tumors. Finally, we summarize the progress of several epigenetic drugs targeting SETD2/H3K36me3, including leukemia and lung cancer. A comprehensive understanding of the role of SETD2-H3K36me3 in the occurrence and development of various tumors is of great significance for future disease diagnosis and treatment.

## 2 SETD2/H3K36ME3

**FIGURE 2:**
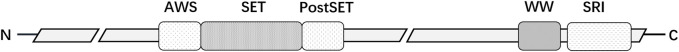
The main domains of SETD2. (1) AWS: Associate with SET domain; (2) SET; Su (var)three to nine, enhancer of zest and trithorax domain; (3) The POST-SET domain and AWS domain are located on both sides of the SET domain to form the AWS- Set-POST SET triple domain; (4) The WW domain is located at the C-terminal; (5) SRI (Set2Rpb1 Interacting) domain is at the end of SETD2 C-terminal.

SETD2 is a methyltransferase that mediates the specific addition of a methyl group to dimethylated lysine-36 of histone H3 (H3K36me2), or three methyl groups to unmethylated lysine-36 of histone H3 (H3K36me3), which interacts with RNA polymerase II to mediate transcription elongation and mismatch repair (Seervai et al., 2020). The SETD2 protein consists of three domains that contribute to its enzymatic function: a AWS-SET-PostSET domain, a WW domain and a Set2 Rpb1-interacting (SRI) domain (Figure 2). The SET domain is located between amino acids 1,550 and 1,667 and is responsible for the activity of histone methyltransferases. The WW domain (amino acids 2,391–2,420) interacts with proline-containing gene sequences and mediates the interaction of Set2 with proteins responsible for the methylation of non-histone substrates (Xie et al., 2008). The SRI domain interacts specifically with the hyperphosphorylated C-terminal domain (CTD) of the largest subunit of RNA Pol II, Rpb1, to regulate Ser2 phosphorylation (Li J et al., 2016).

By mediating the formation of H3K36me3, SETD2 plays important roles in molecular biological processes such as maintaining genome stability, chromatin conformation, gene transcription initiation and elongation (Park et al., 2016). ASH1L is also involved in H3K36 methylation. Although both ASH1L and SETD2 are involved in H3K36 monomethylation and dimethylation, only SETD2 has the potential for trimethylation of H3K36 *in vitro* (Rogawski DS and Cierpicki, 2016). Cells with abnormal SETD2 function often exhibit genomic microsatellite instability and increased frequency of spontaneous mutations, leading to tumorigenesis (Roberti et al., 2016).

## 3 Relationship between tumors and environment

Tumor generation is the result of the interaction of various risk factors, including environmental, exogenous, and endogenous factors, as well as individual factors, including genetic susceptibility (Lewandowska et al., 2019). The environment includes all non-genetic factors such as diet, lifestyle and infectious factors. Broadly speaking, the environment is involved in the causation of most human cancers. The most important environmental factors include outdoor and indoor air pollution as well as soil and drinking water pollution (Boffetta P, 2003). Harmful stimuli from the environment lead to cellular dysfunction, and the accumulation of genetic and epigenetic changes in cells, manifested as the accumulation of chromosomal or molecular aberrations, leading to genetic instability and tumorigenesis.

Tobacco is a very typical example of the relationship between the environment and tumors. Epidemiological studies have clearly confirmed that tobacco can cause various types of cancer. Smoking increases the risk of lung cancer of all histologic types, including squamous cell carcinoma, small cell carcinoma, adenocarcinoma (including bronchiolar/alveolar carcinoma), and large cell carcinoma. In addition to lung cancer, smoking increases the risk of cancers of the oral cavity, larynx, oropharynx, hypopharynx, nasopharynx, esophagus (including squamous cell and adenocarcinoma), stomach, liver, pancreas, bladder, kidney cancer, cervical cancer, and myeloid leukemia (Bettcher and Sanda, 2008; Husain et al., 2021). Similar to tobacco, a growing body of evidence identifies alcohol as an important environmental risk factor for carcinogenesis, and animal experiments support that ethanol is a co-carcinogen and/or tumor promoter under certain conditions. According to the latest research, even a small dose of alcohol has a strong carcinogenic effect, especially in people who smoke at the same time, due to the increased solubility of carcinogens in tobacco smoke in ethanol (Poschl and Seitz, 2004). Nutrients in the diet are also associated with cancer, and nutritional obesity may be associated with recurrence or mortality from the primary cancer.

## 4 The relationship between tumor and epigenetics

Epigenetic modifications include DNA methylation, histone modification, chromatin remodeling and non-coding RNAs (non-coding RNAs, ncRNAs), which play a wide range of roles in regulating gene expression (Castel and Martienssen, 2013). Thus, epigenetics has become an increasingly attractive area of research in recent years.

### 4.1 DNA methylation

DNA methylation refers to the biological process of transferring the methyl group of S-adenosylmethionine (SAM) to cytosine or adenine and is mediated by DNA methyltransferases (DNMTs) (Aran et al., 2013). The cytosine component in DNA cytosine phospho-guanine (CpG) dinucleotide (one 5′-cytosine and one 3′-guanine) is a key site of methylation. The CpG dinucleotide is overexpressed in the gene promoter, and its methylation can silence related genes (Papanicolau-Sengos and Aldape, 2022). Compared with non-tumor tissues, cancers have a complex methylation profile, including overall hypomethylation of the genome, but hypermethylation of CpG islands in gene promoter regions (Gama-Sosa et al., 1983). The *c-Myc* gene is generally considered to be a transcription factor of oncogenes (Prendergast and Ziff, 1991). In cancer cells, hypomethylation is often associated with *c-Myc*. In 1984, hypomethylation of *c-Myc* was first demonstrated in cancer cells cultured *in vitro* and was subsequently identified in a variety of malignant tumors, such as hepatocellular carcinoma, leukemia, gastric cancer, and colorectal cancer (Cheah et al., 1984; Guinney et al., 2015).

### 4.2 Histone modification

Histones are the main protein components in eukaryotic chromatin and are divided into five types: H1, H2A, H2B, H3, and H4 (Audia and Campbell, 2016). In eukaryotic cells, two copies of these histones form octamers and 147 bp of DNA is wound around them to form nucleosomes. Histones are prone to post-transcriptional modifications, including acetylation, methylation, phosphorylation, and ubiquitination. Covalent modification of histones plays an important role in chromatin dynamics and transcriptional regulation (Tsukada et al., 2006).

### 4.3 Chromatin remodeling

Mammalian chromatin remodeling complexes can be divided into four major families: SWI/SNF, ISWI, NuRD/Mi-2/CHD, and INO80 (Wang et al., 2007). As the first to be discovered, the SWI/SNF family comprised ATP-dependent chromatin remodeling complexes (CRCs), which regulate functions such as gene expression and DNA replication, and are related to the occurrence of various cancers. SWI/SNF chromatin remodeling factors are associated with increased chromatin accessibility, which can promote nucleosome repositioning in promoter and enhancer regions, transcription factor binding, recruitment of histone-modifying enzymes and regulation of chromatin loops, promoting the interactions between enhancers and promoters (Wu and Roberts, 2013; Vaicekauskaite et al., 2022). Monterde et al. described recurrent alterations of different *SWI/SNF* genes in nearly 20% of lung cancer patients, which were significantly associated with poorer prognosis, suggesting that *SWI/SNF* genes plays an important role in lung cancer (Monterde and Varela, 2022).

### 4.4 Non-coding RNAs

Non-coding RNAs (ncRNAs) are functional RNA molecules that are not translated into proteins. Based on their length, shape, and location, ncRNAs in cancer have been classified into four major types with distinct functions: microRNA (miRNA), long ncRNA (lncRNA), circular RNA (circRNA), and PIWI interacting RNA (piRNA). A large body of evidence indicates that ncRNAs can act as oncogenes or suppressors to regulate cancer initiation and progression. Many ncRNAs can be released from cancer cells into blood or urine and serve as diagnostic markers or prognostic indicators (Yan and Bu, 2021). Targeted ncRNA therapy has been evaluated in many clinical trials, and the combination with other therapeutic methods will help to achieve better therapeutic effects.

## 5 The relationship between SETD2/H3K36ME3 and various tumors

### 5.1 Renal cell carcinoma

Clear cell renal cell carcinoma (ccRCC) is the most common type of human RCC. The *SETD2* gene is a frequently mutated in renal cancer, especially ccRCC (Li L et al., 2019). In ccRCC, the *PBRM1* gene is often co-mutated with *SETD2* (Varela et al., 2011), and some studies have predicted that *SETD2* mutations are associated with poor prognosis in primary ccRCC (Piva et al., 2015). In RCC, SETD2 participates not only in epigenetic dysfunction, but also in metabolic regulation (Chen et al., 2020). H3K36me3 protein downregulation in the absence of SEDT2 resulted in incomplete gene methylation modification in RCC (Ho et al., 2016). Liu et al. (Liu et al., 2019) found that the loss of *SETD2* downregulated the metabolism of creatine, glycosaminoglycans and carbohydrates via a mechanism that may be related to the peroxisome proliferator-activated receptor γ co-activator 1α (PGC-1α)-mediated metabolic network. This suggests that dysfunction in the SETD2-PGC-1α metabolic pathway in ccRCC may act as a stimulating factor, which provides a new target for drug development and individualized treatment. Wang et al. (Wang J. et al., 2016) showed that low *SETD2* expression was associated with poor prognosis in patients with metastatic RCC (mRCC) treated with tyrosine kinase inhibitors (TKIs), and the frequency of *SETD2* mutation was positively correlated with mRCC progression. This suggested to us that *SETD2* may serve as a potential prognostic biomarker in mRCC patients receiving targeted therapy. However, whether it is applicable to other RCC types requires prospective external validation studies.

### 5.2 Prostate cancer

Prostate cancer is a common malignant tumor in men. Zhang et al. (Zhang et al., 2019) performed Integrated Genome Analysis (IGA) on 51 HMT genes in prostate cancer samples collected from The Cancer Genome Atlas (TCGA) database. The results indicated that the *SETD2* gene may be involved in the androgen receptor response pathway of prostate cancer, and that the *SETD2* gene mutations have a potential role in the occurrence and development of castration-resistant prostate cancer (CRPC). In addition, analysis of mouse models and patient data showed that loss of *SETD2* significantly promotes distant metastasis of prostate cancer. *SETD2* promotes the degradation of EZH2 by methylating the EZH2 K735 site, preventing transformation of cells to a high H3K27me3 chromatin state, thereby inhibiting the molecular mechanism of prostate cancer metastasis. Interestingly, the study also showed that changes in extracellular energy are “sensed” by the AMPK-FOXO3 signaling pathway to regulate *SETD2* expression levels. Furthermore, metformin inhibited the progression of EZH2-high expression prostate cancer by activating the AMPK-FOXO3-SETD2 signaling axis (Yuan et al., 2020). Nevertheless, the potential roles of *SETD2* in the diagnosis, treatment and prognosis of prostate cancer remain to be explored.

### 5.3 Lung adenocarcinoma

Lung cancer is the leading cause of cancer-related deaths in humans, among which lung adenocarcinoma (LUAD) is the main histological subtype with a high mortality rate (Siegel et al., 2023). Hao et al. (Hao et al., 2015) collected surgical specimens from 88 lung cancer patients and established 23 patient-derived xenograft (PDX) models. *SETD2* mutations were identified in both the primary LUAD tissues and the PDX models, with a mutation rate of 21.7%. Walter et al. (Walter et al., 2017) found that loss of *SETD2* and downregulation of H3K36me3 promoted the rapid growth and progression of LUAD. Subsequent studies showed that *SETD2* mutations were significantly associated with poor prognosis (Kadara et al., 2017). These studies suggest that *SETD2* mutations are closely related to the occurrence, development and prognosis of LUAD. Li et al. (Li et al., 2022) retrospectively analyzed the clinical characteristics of 248 Chinese patients with LUAD and found that high tumor mutation burden was significantly associated with high expression of PD-L1. Compared with the PD-L1 low expression group, *SETD2* gene mutations were significantly enriched in the PD-L1 high expression group and correlated with the overall survival of patients. In addition, pathway analysis showed that *SETD2* mutations were involved in the DNA damage repair (DDR) pathway, TP53 pathway, cell cycle pathway and *Drosophila* double wing margin nickeled homologous gene (NOTCH) pathway. The proportions of IFN-γ, CD8^+^ T cells, and NK cells in the *SETD2* mutant group were significantly higher than those in the wild-type group, suggesting that SETD2 may be a potential target for LUAD immunotherapy. The results of *in vitro* and *in vivo* experiments highlighted the ability of SETD2/H3K36me3 to inhibit cell proliferation, migration, invasion and epithelial-mesenchymal transition (EMT) in LUAD by regulating the STAT1-IL‐8 signaling pathway (Yang et al., 2022). Cisplatin is one of the most commonly used chemotherapeutic drugs in the treatment of non-small cell lung cancer (NSCLC), but the mechanism of cisplatin resistance is not fully understood. By high-throughput sequencing of cisplatin-resistant A549 cells selected *in vivo*, Kim et al. (Kim et al., 2019) identified a missense mutation in *SETD2* and showed that SETD2-mediated trimethylation of H3K36 and CREB1 phosphorylation are key targets for cisplatin sensitivity. This study confirms that downregulation of the expression of SETD2 or CREB1 in LUAD cells inhibit the activation of H3K36me3 and ERK, resulting in cisplatin resistance. These studies provide evidence that *SETD2* functions as a tumor suppressor in LUAD and may serve as a novel prognostic biomarker and potential therapeutic target, although in-depth studies are required to elucidate the mechanism.

### 5.4 Nasopharyngeal carcinoma

After knocking out the *SETD2* gene in nasopharyngeal carcinoma (NPC) cells, Zeng et al. detected numerous differentially expressed genes, suggesting that *SETD2* plays an important role in the biological function of NPC. Preliminary classification of the upregulated proteins showed that some were involved in the processes of cell proliferation, adhesion, migration and EMT. Loss of *SETD2* expression in NPC affects 20 classic signaling pathways closely related to tumors, suggesting the potential feasibility of SETD2-targeted therapy (Zeng et al., 2019).

### 5.5 Hepatocellular carcinoma

Using a *SETD2* gene-specific knockout mouse model, Li et al. (Huang et al., 2016) showed that the loss of *SETD2* can lead to spontaneous liver cancer, and can significantly promote diethylnitrosamine (DEN)-induced liver cancer. In DEN-induced liver cancer mouse model, the loss of *SETD2* can significantly increase the number and size of liver tumors. Mechanistic analysis showed that, in addition to regulating the DNA damage response, *SETD2* also inhibited the occurrence of liver cancer by regulating the balance of hepatic lipid metabolism. Loss of *SETD2* resulted in the downregulation of H3K36me3 in lipid efflux-related genes and repressed their expression, which in turn promoted lipid accumulation. Loss of *SETD2* also promoted hepatocarcinogenesis in a high-fat diet-induced model. Chromatin immunoprecipitation sequencing (ChIP-seq) analysis revealed that *SETD2* knockdown induced activation of the c-Jun/activating protein-1 (c-Jun/activating protein-1, c-Jun/AP-1) transcription factor in the liver by promoting lipid accumulation. As an oncogene, c-Jun can inhibit the expression of the p53 gene in *SETD2*-null mice to promote the occurrence of liver cancer. *SETD2* loss promotes the occurrence and development of hepatocellular carcinoma (HCC), and therefore, further studies on the role of *SETD2* and cholesterol homeostasis in tumorigenesis are warranted.

### 5.6 Pancreatic ductal adenocarcinoma

*SETD2* functions as a tumor suppressor in different stages of pancreatic carcinogenesis, and SETD2/H3K36me3 simultaneously inhibits acinar-ductal reprogramming and EMT. Furthermore, loss of SETD2-H3K36me3 affects pancreatic size and acinar cell homeostasis, promoting Kras-induced pancreatic carcinogenesis and metastasis (Niu et al., 2020b). However, the role of *SETD2* mutations in TME remodeling and immune evasion is poorly understood. In another study, in the process of pancreatic carcinogenesis, intratumoral *SETD2* deficiency not only participated in the regulation of pancreatic tumor cell fate, but also regulated immune escape by remodeling neutrophils, which may provide potential therapeutic targets for pancreatic cancer patients with *SETD2* mutation or loss (Niu et al., 2023).

### 5.7 Gastric cancer

Studies have shown that the migration, proliferation and invasion abilities of the gastric cancer (GC) cell lines HGC-27 and AGS decreased as the level of *SETD2* expression increased. Low *SETD2* expression was significantly correlated with clinicopathological parameters such as tumor size, TNM stage, and lymph node metastasis. In addition, patients with low *SETD2* expression had a significantly lower 5-year survival rate compared with patients with high *SETD2* expression (Chen et al., 2018). Further studies showed that H3K36me3 levels were reduced in *SETD2* mutants in GI stromal tumors (GISTs), and *SETD2* silencing promoted DNA damage in GIST-T1 cells. Univariate analysis showed that *SETD2* mutation was associated with shorter recurrence-free survival (RFS) in patients with GISTs (Huang et al., 2016). These findings suggested that downregulation of the *SETD2* gene may be significantly related to the development and poor prognosis of GC.

### 5.8 Colorectal cancer

In colorectal cancer (CRC), *SETD2* has been shown to regulate the Wnt signaling pathway in engineered mouse models (GEMs), and *SETD2* loss promoted tumor progression (Yuan et al., 2017). Furthermore, *SETD2* gene inactivation promoted self-renewal and tissue regeneration of intestinal stem or progenitor cells in mouse intestinal epithelium (Zhang et al., 2014). However, the description of *SETD2* in CRC is limited to clinical case reports (Choi et al., 2014; Liu M. et al., 2021). A recent study of the largest cohort to date revealed that the clinical presentation of *SETD2*-mutant CRC was similar to CRC reported in the general population, although it may be more commonly present in the proximal colon. *SETD2*-mutant CRC may also be found in the presence of p53 mutations and abnormal expression of β-catenin, two proteins known to interact to regulate DNA repair. Furthermore, multiple mutations in the *SETD2* gene may be required for the regulation of H3K36 trimethylation, but further studies are needed to confirm the clinical relevance of this observation. The emergence of next-generation sequencing (NGS) will help to clarify the significance of *SETD2* mutations in CRC (Bushara et al., 2023).

### 5.9 Lymphopoietic system tumors

The maintenance of hematopoietic homeostasis depends on the balance between hematopoietic stem cell self-renewal and differentiation, a process regulated by both genetic and epigenetic mechanisms (Rice et al., 2007; Cullen et al., 2014). Accumulating evidence suggests that this homeostasis may be perturbed by mutations in some key regulatory genes, ultimately leading to hematopoietic malignancy (Papaemmanuil et al., 2016). *SETD2* mutations play an important role in the development and treatment of hematologic malignancies (Huether et al., 2014; Mar et al., 2014). Using the *SETD2* gene conditional knockout mouse model, it was found that *SETD2* plays an important role in maintaining the balance between self-renewal and differentiation of hematopoietic stem cells, and *SETD2*-deficient hematopoietic stem cells (HSPCs) can continue to evolve into systemic malignancies (Zhang et al., 2018). This study provided the first animal model that provides evidence of a causal role for *SETD2* loss in tumorigenesis. *SETD2*-deficient HSPC can acquire the ability to overcome growth disadvantage during the latency period, eventually acquiring to the malignant hematopoietic features of myelodysplastic syndrome (MDS) (Zhang et al., 2018). Studies have shown that *SETD2* plays a tumor suppressor role in chronic myeloid leukemia (CML), and *SETD2* loss significantly promotes imatinib resistance and leukemia stem cell enrichment in CML cells. In addition, the demethylase inhibitor JIB‐04 was shown to restore H3K36me3 levels by blocking H3K36me3 demethylation, thus enhancing the effects of chemotherapy (Sheng et al., 2019). After conditional knockout the *SETD2* gene in MLL-AF9 AML mouse model, homozygous *SETD2* loss was found to delay leukemogenesis, whereas heterozygous *SETD2* loss resulted in accelerated disease progression and chemotherapy resistance (Mar et al., 2017; Skucha et al., 2018). Taken together, these studies provide evidence that *SETD2* functions as a tumor suppressor in hematologic malignancies and that targeting the H3K36me3 demethylase may reverse chemotherapy resistance.

### 5.10 Central nervous system malignancy

High-grade gliomas (HGGs) are a type of poorly differentiated, highly aggressive and migratory brain tumors that occur frequently in both adults and children (Greenall et al., 2017). In whole exome sequencing (WES) analysis of 60 children with HGG, Fontebasso et al. (Fontebasso et al., 2013) found that 15% (11/73) of the children had *SETD2* gene mutations that were mainly truncating mutations. *SETD2* mutations were detected in 8% (5/65) of adult HGGs when analyzed in another independent validation cohort. In addition, the study revealed that *SETD2* mutations are common in older children and young adults, and are distributed mainly in the brain hemispheres. Further Western blot analysis showed that the expression level of H3K36me3 in tumor tissues with *SETD2* gene mutations was decreased. In subsequent studies, Huether et al. (Cheah et al., 1984) confirmed that *SETD2* gene mutations could be detected in childhood HGG tissues and glioblastoma cell lines. *SETD2* mutations are also present in low-grade gliomas, mainly in people aged over 55 years (Brennan et al., 2013). These studies suggest that *SETD2* functions as a tumor suppressor in glioma.

### 5.11 Breast cancer

Although there are many treatment methods for breast cancer (BC), including surgery, radiotherapy, chemotherapy, and immunotherapy, the morbidity and mortality remain high (Kashyap et al., 2022). Sarakbi et al. (Al Sarakbi et al., 2009) analyzed the expression of *SETD2* mRNA in a long-term follow-up cohort of breast cancer patients and found that *SETD2* expression levels were significantly reduced in samples from patients who developed metastasis, local recurrence, or died of BC. In addition, *SETD2* expression levels were negatively correlated with tumor stage, grade, and lymph node metastasis. According to TCGA and METABRIC databases, *SETD2* is mutated in 2.62% of all BC subtypes and 1.2% of triple-negative breast cancer cases. However, whether *SETD2* is mutated in other BC subtypes is unknown. Further studies suggested that the expression level of SETD2 was significantly positively correlated with the prognosis of patients. However, *SETD2* mutations have little effect on the outcome of chemotherapy in patients (Morcillo-Garcia et al., 2019). Thus, *SETD2* may function as a tumor suppressor, and may be a potential prognostic marker in BC.

### 5.12 Other systemic tumors

Osteosarcoma (OS) is the most common primary bone malignancy in children and adolescents, and occurs most frequently in the distal femur, tibia, and proximal humerus (Gill and Gorlick, 2021). In 2017, Behjati et al. detected *SETD2* mutations in less than 2% of human osteosarcoma samples. Subsequent WES on osteosarcoma-susceptible dogs indicated that osteosarcoma may originate from *SETD2* mutations, which function as oncogenic drivers (Behjati et al., 2017; Sakthikumar et al., 2018). *SETD2* mutations have also been detected in osteosarcoma, myxoid liposarcoma (MLPS), and synovial sarcoma (SYN) (Sakthikumar et al., 2018), although the effect of *SETD2* on the biological function of osteosarcoma has not yet been reported. Comprehensive genome analysis of 24 choriocarcinoma patients showed that chromatin regulatory genes, particularly *SETD2*, were frequently altered in chordoma (Wang L. et al., 2016). *SETD2* mutations were also reported in 22% (11/50) of patients with malignant peritoneal mesothelioma (MPM) (Offin et al., 2022). These studies suggest that *SETD2* may provide a new therapeutic target for various types of malignancies.

## 6 Advances in epigenetic drug targeting SETD2/H3K36ME3

Epigenetic regulation plays a key role in tumorigenesis and development. Among these factors, HMTs are attractive targets for disease intervention because they are frequently dysregulated in a range of human tumors and their enzymatic activity can be manipulated therapeutically (Baylin and Jones, 2011). Loss of *SETD2* has been reported to enhance the Wnt/β-catenin signaling pathway, thereby affecting intestinal self-renewal and differentiation. Therefore, tumors lacking this methyltransferase exhibit more aggressiveness and poorer prognosis, with potential therapeutic implications (Yuan et al., 2017). It is hoped that integration of data from genomics, transcriptomics and epigenomics studies will facilitate the discovery of relevant epigenetic therapeutic targets in the near future.

As novel oncogenic targets, MYCN and ERG were shown to be direct downstream targets of SETD2. In CML cell lines, *SETD2* knockout-induced overexpression resulted in imatinib insensitivity and enrichment of leukemia stem cells. JIB-04, an inhibitor that restores H3K36me3 levels by blocking H3K36me3 demethylation, successfully increased the sensitivity of lymphohematopoietic tumor cells to imatinib, providing a potential therapeutic strategy (Sheng et al., 2019). Targeting the epigenome is a fairly new approach in lung cancer therapy to address chemotherapy resistance and reverse immune escape (Duruisseaux and Esteller, 2018). Adavosertib (AZD1775), a highly potent inhibitor of WEE1 kinase, is a key regulator of G2/M and S phase checkpoints, which may prevent tumor cell growth by blocking some enzymes required for cell growth (Liu J. F. et al., 2021).

## 7 Summary

In this review, we first summarize the structure and function of the *SETD2* and the role of SETD2-H3K36me3 in mediating the important relationship between the environment and tumors. Changes in *SETD2* lead to abnormal regulation of H3K36me3, thereby promoting tumorigenesis and development. *SETD2* gene mutation or functional loss leads to dysfunction of downstream signaling pathways, including the Wnt signaling and PGC1-α metabolic pathways, and this dysfunction is related to tumorigenesis. In addition, signaling pathways such as the Wnt/β-catenin and ERK signaling pathways are related to drug resistance. *SETD2* is frequently mutated in a wide range of tumor types, suggesting that *SETD2* functions as a tumor suppressor. Furthermore, as a novel molecular therapeutic target, *SETD2* has provided new opportunities in the diagnosis and treatment of acute leukemia. At the same time, the rapid development of high-throughput technology will provide new ideas for the discovery and screening of epigenetic drugs targeting *SETD2*.

In summary, SETD2, as a new tumor suppressor factor, exhibits gene mutation or low protein expression in many human malignant tumors, although the exact mechanism is unclear. There are few reports of the changes in the expression of tumor-related genes caused by *SETD2* gene mutations, and corresponding experimental research, especially *in vitro* studies, is rare. Recent studies have indicated that SETD2 mutations may serve as potential biomarkers for predicting immunotherapy efficacy (Lu et al., 2021). In-depth investigations of the role of *SETD2* in the process of tumor formation and development and the underlying mechanism are of great significance for the diagnosis, treatment and prevention of tumors.

## Figures and Tables

**Table:** 

**SETD2**	SET domain-containing 2
**SAM**	S-adenosylmethionine
**DNMTs**	DNA metlhyltransferases
**CpG**	Cytosine phospho-guanine
**CRCs**	Chromatin remodeling complexes
**ncRNAs**	Non-coding RNAs
**lncRNA**	Long ncRNA
**circRNA**	Circular RNA
**piRNA**	PIWI interacting RNA
**ccRCC**	Clear cell renal cell carcinoma
**PGC-1α**	Peroxisome proliferator-activated receptor γ co-activator 1α
**mRCC**	Metastatic renal cell carcinoma
**TCGA**	The cancer genome atlas
**HMT**	Histone methyltransferase
**CRPC**	Castration-resistant prostate cancer
**LUAD**	Lung adenocarcinoma
**PDXs**	Patient-derived xenografts
**DDR**	DNA damage repair pathway
**NOTCH**	*Drosophila* double wing margin nicked homologous gene
**EMT**	Epithelial-mesenchymal transition
**NSCLC**	Non-small cell lung cancer
**NPC**	Nasopharyngeal carcinoma
**DEN**	Diethylnitrosamine
**c-Jun/AP-1**	C-Jun/activating protein-1
**HCC**	Hepatocellular carcinoma
**PDAC**	Pancreatic ductal adenocarcinoma
**GC**	Gastric cancer
**GISTs**	GI stromal tumors
**RFS**	Relapse-free survival
**CRC**	Colorectal cancer
**GEMs**	Engineered mouse models
**NGS**	Next-generation sequencing
**HSPCs**	Hematopoietic stem/progenitor cells
**MDS**	Myelodysplastic syndrome
**CML**	Chronic myeloid leukemia
**HGGs**	High-grade gliomas
**BC**	Breast cancer
**OS**	Osteosarcoma
**WES**	Whole exome sequencing
**MLPS**	Myxoid liposarcoma
**SYN**	Synovial sarcoma
**MPeM**	Malignant peritoneal mesothelioma
**HMTs**	Histone methyltransferases

## References

[B1] Al Sarakbi W. Sasi W. Jiang W. G. Roberts T. Newbold R. F. Mokbel K. (2009). The mRNA expression of SETD2 in human breast cancer: The mRNA expression of SETD2 in human breast cancer: correlation with clinico-pathological parametersorrelation with clinico-pathological parameters. BMC Cancer 9, 290. 10.1186/1471-2407-9-290 19698110 PMC3087337

[B2] Aran D. Sabato S. Hellman A. (2013). DNA methylation of distal regulatory sites characterizes dysregulation of cancer genes. Genome Biol. 14 (3), R21. 10.1186/gb-2013-14-3-r21 23497655 PMC4053839

[B3] Audia J. E. Campbell R. M. (2016). Histone Histone Modifications and Cancerodifications and cancer. Cold Spring Harb. Perspect. Biol. 8 (4), a019521. 10.1101/cshperspect.a019521 27037415 PMC4817802

[B4] Baylin S. B. Jones P. A. (2011). A decade of exploring the cancer epigenome - biological and translational implications. Nat. Rev. Cancer 11 (10), 726–73434. 10.1038/nrc3130 21941284 PMC3307543

[B5] Behjati S. Tarpey P. S. Haase K. Ye H. Young M. D. Alexandrov L. B. (2017). Recurrent mutation of IGF signalling genes and distinct patterns of genomic rearrangement in osteosarcoma. Nat. Commun. 8, 15936. 10.1038/ncomms15936 28643781 PMC5490007

[B6] Bettcher D. W. Sanda L. S. (2008). Clinical cancer control and prevention. Eliminating tobacco-induced cancers: Clinical cancer control and prevention. Eliminating tobacco-induced cancers: a worldwide challenge worldwide challenge. Ann. Oncol. 19 (7), vii230–3. 10.1093/annonc/mdn435 18790957

[B7] Boffetta P N. F. (2003). Contribution of environmental factors to cancer risk. 10.1093/bmp/ldg023 14757710

[B8] Brennan C. W. Verhaak R. G. McKenna A. Campos B. Noushmehr H. Salama S. R. (2013). The somatic genomic landscape of glioblastoma. Cell 155 (2), 462–47777. 10.1016/j.cell.2013.09.034 24120142 PMC3910500

[B9] Bushara O. Wester J. R. Jacobsen D. Sun L. Weinberg S. Gao J. (2023). Clinical and histopathologic characterization of SETD2-mutated colorectal cancer. Hum. Pathol. 131, 9–16. 10.1016/j.humpath.2022.12.001 36502925 PMC9875556

[B10] Cancer N. (2013). Genome Atlas Research, Comprehensive molecular characterization of clear cell renal cell carcinoma. Nature 499 (7456), 43–49. 23792563 10.1038/nature12222 PMC3771322

[B11] Castel S. E. Martienssen R. A. (2013). RNA interference in the nucleus: RNA interference in the nucleus: roles for small RNAs in transcription, epigenetics and beyondoles for small RNAs in transcription, epigenetics and beyond. Nat. Rev. Genet. 14 (2), 100–112. 10.1038/nrg3355 23329111 PMC4205957

[B12] Cheah M. S. Wallace C. D. Hoffman R. M. (1984). Hypomethylation of DNA in human cancer cells: Hypomethylation of DNA in human cancer cells: a site-specific change in the c-myc oncogene site-specific change in the c-myc oncogene. J. Natl. Cancer Inst. 73 (5), 1057–1065. 6092764

[B13] Chen R. Zhao W. Q. Fang C. Yang X. Ji M. (2020). Histone methyltransferase SETD2: Histone methyltransferase SETD2: a potential tumor suppressor in solid cancers potential tumor suppressor in solid cancers. J. Cancer 11 (11), 3349–3356. 10.7150/jca.38391 32231741 PMC7097956

[B14] Chen Z. Raghoonundun C. Chen W. Zhang Y. Tang W. Fan X. (2018). SETD2 indicates favourable prognosis in gastric cancer and suppresses cancer cell proliferation, migration, and invasion. Biochem. Biophys. Res. Commun. 498 (3), 579–585. 10.1016/j.bbrc.2018.03.022 29522714

[B15] Choi Y. J. Oh H. R. Choi M. R. Gwak M. An C. H. Chung Y. J. (2014). Frameshift mutation of a histone methylation-related gene SETD1B and its regional heterogeneity in gastric and colorectal cancers with high microsatellite instability. Hum. Pathol. 45 (8), 1674–1681. 10.1016/j.humpath.2014.04.013 24925220

[B16] Cullen S. M. Mayle A. Rossi L. Goodell M. A. (2014). Hematopoietic stem cell development: Hematopoietic stem cell development: an epigenetic journeyn epigenetic journey. Curr. Top. Dev. Biol. 107, 39–75. 10.1016/B978-0-12-416022-4.00002-0 24439802

[B17] Dong Y. Zhao X. Feng X. Zhou Y. Yan X. Zhang Y. (2019). SETD2 mutations confer chemoresistance in acute myeloid leukemia partly through altered cell cycle checkpoints. Leukemia 33 (11), 2585–2598. 10.1038/s41375-019-0456-2 30967619 PMC6785365

[B18] Duruisseaux M. Esteller M. (2018). Lung cancer epigenetics: From knowledge to applications. Semin. Cancer Biol. 51, 116–128. 10.1016/j.semcancer.2017.09.005 28919484

[B19] Fontebasso A. M. Schwartzentruber J. Khuong-Quang D. A. Liu X. Y. Sturm D. Korshunov A. (2013). Mutations in SETD2 and genes affecting histone H3K36 methylation target hemispheric high-grade gliomas. Acta Neuropathol. 125 (5), 659–66969. 10.1007/s00401-013-1095-8 23417712 PMC3631313

[B20] Gama-Sosa S. V. M. A. Trewyn R. W. Oxenhandler R. Kuo K. C. Gehrke C. W. Ehrlich M. (1983). The 5-methylcytosine content of DNA from human tumors. Nucleic Acids Res 11, 6883–6894. 10.1093/nar/11.19.6883 6314264 PMC326421

[B21] Gill J. Gorlick R. (2021). Advancing therapy for osteosarcoma. Nat. Rev. Clin. Oncol. 18 (10), 609–624. 10.1038/s41571-021-00519-8 34131316

[B22] Greenall S. A. Lim Y. C. Mitchell C. B. Ensbey K. S. Stringer B. W. Wilding A. L. (2017). Cyclin-dependent kinase 7 is a therapeutic target in high-grade glioma. Oncogenesis 6 (5), e336. 10.1038/oncsis.2017.33 28504693 PMC5523066

[B23] Guinney J. Dienstmann R. Wang X. de Reynies A. Schlicker A. Soneson C. (2015). The consensus molecular subtypes of colorectal cancer. Nat. Med. 21 (11), 1350–13566. 10.1038/nm.3967 26457759 PMC4636487

[B24] Hao C. Wang L. Peng S. Cao M. Li H. Hu J. (2015). Gene mutations in primary tumors and corresponding patient-derived xenografts derived from non-small cell lung cancer. Cancer Lett. 357 (1), 179–185. 10.1016/j.canlet.2014.11.024 25444907 PMC4301580

[B25] Ho T. H. Park I. Y. Zhao H. Tong P. Champion M. D. Yan H. (2016). High-resolution profiling of histone h3 lysine 36 trimethylation in metastatic renal cell carcinoma. Oncogene 35 (12), 1565–157474. 10.1038/onc.2015.221 26073078 PMC4679725

[B26] Huang K. K. McPherson J. R. Tay S. T. Das K. Tan I. B. Ng C. C. (2016). SETD2 histone modifier loss in aggressive GI stromal tumours. Gut 65 (12), 1960–1972. 10.1136/gutjnl-2015-309482 26338826

[B27] Huether R. Dong L. Chen X. Wu G. Parker M. Wei L. (2014). The landscape of somatic mutations in epigenetic regulators across 1,000 paediatric cancer genomes. Nat. Commun. 5, 3630. 10.1038/ncomms4630 24710217 PMC4119022

[B28] Husain M. J. Datta B. K. Nargis N. Iglesias R. Perucic A. M. Ahluwalia I. B. (2021). Revisiting the association between worldwide implementation of the MPOWER package and smoking prevalence, 2008-2017. Tob. Control 30 (6), 630–637. 10.1136/tobaccocontrol-2020-055758 32893187 PMC8543233

[B29] Kadara H. Choi M. Zhang J. Parra E. R. Rodriguez-Canales J. Gaffney S. G. (2017). Whole-exome sequencing and immune profiling of early-stage lung adenocarcinoma with fully annotated clinical follow-up. Ann. Oncol. 28 (1), 75–82. 10.1093/annonc/mdw436 27687306 PMC5982809

[B30] Kashyap D. Pal D. Sharma R. Garg V. K. Goel N. Koundal D. (2022). Global Global Increase in Breast Cancer Incidence: Risk Factors and Preventive Measuresncrease in breast cancer incidence: Risk factors and preventive measures. Biomed. Res. Int. 2022, 9605439. 10.1155/2022/9605439 35480139 PMC9038417

[B31] Kim I.-K. McCutcheon J. N. Rao G. Liu S. V. Pommier Y. Skrzypski M. (2019). Acquired SETD2 mutation and impaired CREB1 activation confer cisplatin resistance in metastatic non-small cell lung cancer. Oncogene 38 (2), 180–193. 10.1038/s41388-018-0429-3 30093630 PMC8274951

[B32] Leung W. Teater M. Durmaz C. Meydan C. Chivu A. G. Chadburn A. (2022). SETD2 SETD2 Haploinsufficiency Enhances Germinal Center-Associated AICDA Somatic Hypermutation to Drive B-cell Lymphomagenesisaploinsufficiency enhances germinal center-associated AICDA somatic hypermutation to drive B-cell lymphomagenesis. Cancer Discov. 12 (7), 1782–1803. 10.1158/2159-8290.CD-21-1514 35443279 PMC9262862

[B33] Lewandowska A. M. Rudzki M. Rudzki S. Lewandowski T. Laskowska B. (2019). Environmental risk factors for cancer - review paper. Ann. Agric. Environ. Med. 26 (1), 1–7. 10.26444/aaem/94299 30922021

[B34] Li J D. G. Westers H. Sijmons R. van den Berg A. Kok K. (2016). SETD2: An epigenetic modifier with tumor suppressor functionality. 10.18632/oncotarget.9368 PMC5226616 27191891

[B35] Li K. Liu J. Wu L. Xiao Y. Li J. Du H. (2022). Genomic correlates of programmed cell death ligand 1 (PD-L1) expression in Chinese lung adenocarcinoma patients. Cancer Cell Int. 22 (1), 138. 10.1186/s12935-022-02488-z 35346207 PMC8962080

[B36] Li L M. W. Huang M. Williams P. Wang Y. (2019). Integrated genomic and proteomic analyses reveal novel mechanisms of the methyltransferase SETD2 in renal cell carcinoma development. Mol. Cell Proteomics. 10.1074/mcp.RA118.000957 PMC6398210 30487242

[B37] Liu J. F. Xiong N. Campos S. M. Wright A. A. Krasner C. Schumer S. (2021b). Phase II Phase II Study of the WEE1 Inhibitor Adavosertib in Recurrent Uterine Serous Carcinomatudy of the WEE1 inhibitor adavosertib in recurrent uterine serous carcinoma. J. Clin. Oncol. Official J. Am. Soc. Clin. Oncol. 39 (14), 1531–1539. 10.1200/JCO.20.03167 33705205

[B38] Liu J. Hanavan P. D. Kras K. Ruiz Y. W. Castle E. P. Lake D. F. (2019). Loss of SETD2 Loss of SETD2 Induces a Metabolic Switch in Renal Cell Carcinoma Cell Lines toward Enhanced Oxidative Phosphorylationnduces a metabolic switch in renal cell carcinoma cell lines toward enhanced oxidative phosphorylation. J. Proteome Res. 18 (1), 331–340. 10.1021/acs.jproteome.8b00628 30406665 PMC6465098

[B39] Liu M. Rao H. Liu J. Li X. Feng W. Gui L. (2021a). The histone methyltransferase SETD2 modulates oxidative stress to attenuate experimental colitis. Redox Biol. 43, 102004. 10.1016/j.redox.2021.102004 34020310 PMC8141928

[B40] Lu M. Zhao B. Liu M. Wu L. Li Y. Zhai Y. (2021). Pan-cancer analysis of SETD2 mutation and its association with the efficacy of immunotherapy. NPJ Precis. Oncol. 5 (1), 51. 10.1038/s41698-021-00193-0 34127768 PMC8203790

[B41] Mar B. G. Bullinger L. B. McLean K. M. Grauman P. V. Harris M. H. Stevenson K. (2014). Mutations in epigenetic regulators including SETD2 are gained during relapse in paediatric acute lymphoblastic leukaemia. Nat. Commun. 5, 3469. 10.1038/ncomms4469 24662245 PMC4016990

[B42] Mar B. G. Chu S. H. Kahn J. D. Krivtsov A. V. Koche R. Castellano C. A. (2017). SETD2 alterations impair DNA damage recognition and lead to resistance to chemotherapy in leukemia. Blood 130 (24), 2631–2641. 10.1182/blood-2017-03-775569 29018079 PMC5731084

[B43] Monterde B. Varela I. (2022). Role of SWI/SNF chromatin remodeling genes in lung cancer development. Biochem. Soc. Trans. 50 (3), 1143–1150. 10.1042/BST20211084 35587173

[B44] Morcillo-Garcia S. Noblejas-Lopez M. D. M. Nieto-Jimenez C. Perez-Pena J. Nuncia-Cantarero M. Gyorffy B. (2019). Genetic mutational status of genes regulating epigenetics: Role of the histone methyltransferase KMT2D in triple negative breast tumors. PLoS One 14 (4), e0209134. 10.1371/journal.pone.0209134 30990809 PMC6467442

[B45] Niu N. Lu P. Yang Y. He R. Zhang L. Shi J. (2020b). Loss of Setd2 promotes Kras-induced acinar-to-ductal metaplasia and epithelia-mesenchymal transition during pancreatic carcinogenesis. Gut 69 (4), 715–726. 10.1136/gutjnl-2019-318362 31300513

[B46] Niu N. Lu P. Yang Y. He R. Zhang L. Shi J. (2020a). Loss of Setd2 promotes Kras-induced acinar-to-ductal metaplasia and epithelia-mesenchymal transition during pancreatic carcinogenesis. Gut 69 (4), 715–726. 10.1136/gutjnl-2019-318362 31300513

[B47] Niu N. Shen X. Zhang L. Chen Y. Lu P. Yang W. (2023). Tumor Tumor Cell-Intrinsic SETD2 Deficiency Reprograms Neutrophils to Foster Immune Escape in Pancreatic Tumorigenesisell-intrinsic SETD2 deficiency reprograms neutrophils to foster immune escape in pancreatic tumorigenesis. Adv. Sci. (Weinh) 10 (2), e2202937. 10.1002/advs.202202937 36453584 PMC9839845

[B48] Offin M. Yang S. R. Egger J. Jayakumaran G. Spencer R. S. Lopardo J. (2022). Molecular Molecular Characterization of Peritoneal Mesotheliomasharacterization of peritoneal mesotheliomas. J. Thorac. Oncol. 17 (3), 455–460. 10.1016/j.jtho.2021.09.012 34648949 PMC8882128

[B49] Papaemmanuil E. Gerstung M. Bullinger L. Gaidzik V. I. Paschka P. Roberts N. D. (2016). Genomic Genomic Classification and Prognosis in Acute Myeloid Leukemialassification and prognosis in acute myeloid leukemia. N. Engl. J. Med. 374 (23), 2209–2221. 10.1056/NEJMoa1516192 27276561 PMC4979995

[B50] Papanicolau-Sengos A. Aldape K. (2022). DNA DNA Methylation Profiling: An Emerging Paradigm for Cancer Diagnosisethylation profiling: An emerging paradigm for cancer diagnosis. Annu. Rev. Pathol. 17, 295–321. 10.1146/annurev-pathol-042220-022304 34736341

[B51] Park I. Y. Powell R. T. Tripathi D. N. Dere R. Ho T. H. Blasius T. L. (2016). Dual Dual Chromatin and Cytoskeletal Remodeling by SETD2hromatin and cytoskeletal remodeling by SETD2. Cell 166 (4), 950–962. 10.1016/j.cell.2016.07.005 27518565 PMC5101839

[B52] Piva F. Santoni M. Matrana M. R. Satti S. Giulietti M. Occhipinti G. (2015). BAP1, PBRM1 and SETD2 in clear-cell renal cell carcinoma: BAP1, PBRM1 and SETD2 in clear-cell renal cell carcinoma: molecular diagnostics and possible targets for personalized therapiesolecular diagnostics and possible targets for personalized therapies. Expert Rev. Mol. Diagn 15 (9), 1201–121010. 10.1586/14737159.2015.1068122 26166446

[B53] Poschl G. Seitz H. K. (2004). Alcohol and cancer. Alcohol Alcohol 39 (3), 155–16565. 10.1093/alcalc/agh057 15082451

[B54] Prendergast G. C. Ziff E. B. (1991). Methylation-sensitive sequence-specific DNA binding by the c-Myc basic region. Science 251 (4990), 186–189. 10.1126/science.1987636 1987636

[B55] Rice K. L. Hormaeche I. Licht J. D. (2007). Epigenetic regulation of normal and malignant hematopoiesis. Oncogene 26 (47), 6697–6714714. 10.1038/sj.onc.1210755 17934479

[B56] Roberti A. Dobay M. P. Bisig B. Vallois D. Boéchat C. Lanitis E. (2016). Type II enteropathy-associated T-cell lymphoma features a unique genomic profile with highly recurrent SETD2 alterations. Nat. Commun. 7, 12602. 10.1038/ncomms12602 27600764 PMC5023950

[B57] Rogawski Ds G. J. Cierpicki T. (2016). H3K36 methyltransferases as cancer drug targets: Rationale and perspectives for inhibitor development. Future Med. Chem. Future Med. Chem. 10.4155/fmc-2016-0071 PMC5020427 27548565

[B58] Sakthikumar S. Elvers I. Kim J. Arendt M. L. Thomas R. Turner-Maier J. (2018). SETD2 SETD2 Is Recurrently Mutated in Whole-Exome Sequenced Canine Osteosarcomas recurrently mutated in whole-exome sequenced canine osteosarcoma. Cancer Res. 78 (13), 3421–3431. 10.1158/0008-5472.CAN-17-3558 29724721

[B59] Seervai R. N. H. Jangid R. K. Karki M. Tripathi D. N. Jung S. Y. Kearns S. E. (2020). The Huntingtin-interacting protein SETD2/HYPB is an actin lysine methyltransferase. Sci. Adv. 6 (40), eabb7854. 10.1126/sciadv.abb7854 33008892 PMC7852384

[B60] Sheng Y. Ji Z. Zhao H. Wang J. Cheng C. Xu W. (2019). Downregulation of the histone methyltransferase SETD2 promotes imatinib resistance in chronic myeloid leukaemia cells. Cell Prolif. 52 (4), e12611. 10.1111/cpr.12611 31054182 PMC6668982

[B61] Siegel R. L. Miller K. D. Wagle N. S. Jemal A. (2023). Cancer statistics, 2023. a Cancer J. For Clin. 73 (1), 17–48. 10.3322/caac.21763 36633525

[B62] Skucha A. Ebner J. Grebien F. (2018). SETD2 in MLL-rearranged leukemia - a complex case. Mol. Cell Oncol. 5 (4), e1503492. 10.1080/23723556.2018.1503492 30250934 PMC6150042

[B63] Sung H. Ferlay J. Siegel R. L. Laversanne M. Soerjomataram I. Jemal A. (2021). Global Global Cancer Statistics 2020: GLOBOCAN Estimates of Incidence and Mortality Worldwide for 36 Cancers in 185 Countriesancer statistics 2020: GLOBOCAN estimates of incidence and mortality worldwide for 36 cancers in 185 countries. CA a Cancer J. For Clin. 71 (3), 209–249. 10.3322/caac.21660 33538338

[B64] Tsukada Y. Fang J. Erdjument-Bromage H. Warren M. E. Borchers C. H. Tempst P. (2006). Histone demethylation by a family of JmjC domain-containing proteins. Nature 439 (7078), 811–8166. 10.1038/nature04433 16362057

[B65] Vaicekauskaite I. Sabaliauskaite R. Lazutka J. R. Jarmalaite S. (2022). The The Emerging Role of Chromatin Remodeling Complexes in Ovarian Cancermerging role of chromatin remodeling complexes in ovarian cancer. Int. J. Mol. Sci. 23 (22), 13670. 10.3390/ijms232213670 36430148 PMC9697406

[B66] Varela I. Tarpey P. Raine K. Huang D. Ong C. K. Stephens P. (2011). Exome sequencing identifies frequent mutation of the SWI/SNF complex gene PBRM1 in renal carcinoma. Nature 469 (7331), 539–54242. 10.1038/nature09639 21248752 PMC3030920

[B67] Walter D. M. Venancio O. S. Buza E. L. Tobias J. W. Deshpande C. Gudiel A. A. (2017). Systematic Systematic *In Vivo* Inactivation of Chromatin-Regulating Enzymes Identifies Setd2 as a Potent Tumor Suppressor in Lung Adenocarcinoman vivo inactivation of chromatin-regulating enzymes identifies Setd2 as a potent tumor suppressor in lung adenocarcinoma. Cancer Res. 77 (7), 1719–1729. 10.1158/0008-5472.CAN-16-2159 28202515 PMC5380596

[B68] Wang G. G. Allis C. D. Chi P. (2007). Chromatin remodeling and cancer, Part II: ATP-dependent chromatin remodeling. Trends Mol. Med. 13 (9), 373–38080. 10.1016/j.molmed.2007.07.004 17822959 PMC4337864

[B69] Wang J. Liu L. Qu Y. Xi W. Xia Y. Bai Q. (2016a). Prognostic Prognostic Value of SETD2 Expression in Patients with Metastatic Renal Cell Carcinoma Treated with Tyrosine Kinase Inhibitorsalue of SETD2 expression in patients with metastatic renal cell carcinoma treated with tyrosine kinase inhibitors. J. Urol. 196 (5), 1363–1370. 10.1016/j.juro.2016.06.010 27288695

[B70] Wang L. Zehir A. Nafa K. Zhou N. Berger M. F. Casanova J. (2016b). Genomic aberrations frequently alter chromatin regulatory genes in chordoma. Genes Chromosom. Cancer 55 (7), 591–600. 10.1002/gcc.22362 27072194 PMC5031498

[B71] Wu J. N. Roberts C. W. (2013). ARID1A mutations in cancer: Another epigenetic tumor suppressor? Cancer Discov. 3 (1), 35–43. 10.1158/2159-8290.CD-12-0361 23208470 PMC3546152

[B72] Xie P. Tian C. An L. Nie J. Lu K. Xing G. (2008). Histone methyltransferase protein SETD2 interacts with p53 and selectively regulates its downstream genes. Cell Signal 20 (9), 1671–1678. 10.1016/j.cellsig.2008.05.012 18585004

[B73] Yan H. Bu P. (2021). Non-coding RNA in cancer. Essays Biochem. 65 (4), 625–639. 10.1042/EBC20200032 33860799 PMC8564738

[B74] Yang X. Chen R. Chen Y. Zhou Y. Wu C. Li Q. (2022). Methyltransferase SETD2 inhibits tumor growth and metastasis via STAT1-IL-8 signaling-mediated epithelial-mesenchymal transition in lung adenocarcinoma. Cancer Sci. 113 (4), 1195–1207. 10.1111/cas.15299 35152527 PMC8990294

[B75] Yuan H. Han Y. Wang X. Li N. Liu Q. Yin Y. (2020). SETD2 SETD2 Restricts Prostate Cancer Metastasis by Integrating EZH2 and AMPK Signaling Pathwaysestricts prostate cancer metastasis by integrating EZH2 and AMPK signaling pathways. Cancer Cell 38 (3), 350–365. 10.1016/j.ccell.2020.05.022 32619406

[B76] Yuan H. Li N. Fu D. Ren J. Hui J. Peng J. (2017). Histone methyltransferase SETD2 modulates alternative splicing to inhibit intestinal tumorigenesis. J. Clin. Investigation 127 (9), 3375–3391. 10.1172/JCI94292 PMC5669571 28825595

[B77] Zeng Y. Wang S. Feng M. Shao Z. Yuan J. Shen Z. (2019). Quantitative proteomics and differential signal enrichment in nasopharyngeal carcinoma cells with or without SETD2 gene knockout. Nan Fang. Yi Ke Da Xue Xue Bao 39 (10), 1191–1199. 10.12122/j.issn.1673-4254.2019.10.10 31801714 PMC6867956

[B78] Zhang Y. L. Sun J. W. Xie Y. Y. Zhou Y. Liu P. Song J. C. (2018). Setd2 deficiency impairs hematopoietic stem cell self-renewal and causes malignant transformation. Cell Res. 28 (4), 476–490. 10.1038/s41422-018-0015-9 29531312 PMC5939047

[B79] Zhang Y. Xie S. Zhou Y. Xie Y. Liu P. Sun M. (2014). H3K36 H3K36 Histone Methyltransferase Setd2 Is Required for Murine Embryonic Stem Cell Differentiation toward Endodermistone methyltransferase Setd2 is required for murine embryonic stem cell differentiation toward endoderm. Cell Rep. 9 (3), 1171. 10.1016/j.celrep.2014.10.050 25242323

[B80] Zhang Y. Yan L. Yao W. Chen K. Xu H. Ye Z. (2019). Integrated Integrated Analysis of Genetic Abnormalities of the Histone Lysine Methyltransferases in Prostate Cancernalysis of genetic abnormalities of the histone lysine methyltransferases in prostate cancer. Med. Sci. Monit. 25, 193–239. 10.12659/msm.912294 30616239 PMC6330996

[B81] Zhou Y Z. X. Xu B. Deng H. Chen L. Jiang J. (2020). Histone methyltransferase SETD2 inhibits tumor growth via suppressing CXCL1-mediated activation of cell cycle in lung adenocarcinoma. Aging (Albany NY) 12 (24), 25189–25206. 10.18632/aging.104120 33223508 PMC7803529

